# Program

**DOI:** 10.1111/jdi.12685

**Published:** 2017-05-05

## Opening Ceremony

### Friday, May 19 7:40˜8:00 Reception Hall (West), Nagoya Congress Center Bldg. 1, 4F

Jiro Nakamura

60th JDS President / 9th AASD President, Japan

Yutaka Seino

AASD Chair, Japan

Takashi Kadowaki

JDS Chairman, Japan

## The Yutaka Seino Distinguished Leadership Award Lecture

### Friday, May 19 11:00˜11:30 Reception Hall (West), Nagoya Congress Center Bldg. 1, 4F

ChairHong Kyu LeeDepartment of Internal Medicine, Seoul National University, Korea

A‐AL1

**A journey following the track of my international works and research activities**

Nigishi Hotta

Professor Emeritus Nagoya University / Director General Emeritus Japan Organization of Occupational Health and safety CHUBU ROSAI HOSPITAL, Japan

## The Masato Kasuga Award for Outstanding Scientic Achievement Lecture

### Friday, May 19 13:00˜13:30 Reception Hall (West), Nagoya Congress Center Bldg. 1, 4F

ChairMasato KasugaNational Center for Global Health and Medicine, Japan

A‐AL2

**Incretins and incretin‐related drugs in the management of type 2 diabetes: From Asian perspectives**

Daisuke Yabe

Kansai Electric Power Medical Research Institute and Kyoto University Graduate School of Medicine, Japan

## The Xiaoren Pan Distinguished Research Award for Epidemiology of Diabetes in Asia

### Saturday, May 20 13:10˜13:40 Reception Hall (West), Nagoya Congress Center Bldg. 1, 4F

ChairLee‐Ming ChuangDepartment of Internal Medicine, National Taiwan University Medical College, Taiwan

A‐AL3

**Prognosis, diagnosis and prevention of diabetes in China**

Weiping Jia

Professor, President of Chinese Diabetes Society / Director of Shanghai Jiao Tong University Affiliated Sixth People's Hospital, China

## Presidential Lecture

### Saturday, May 20 8:20˜8:50 Reception Hall (West), Nagoya Congress Center Bldg. 1, 4F

ChairYutaka SeinoKansai Electric Power Hospital / Kansai Electric Power Medical Research Institute, Japan

A‐PL

**Current and future strategies for treatment of diabetic neuropathy**

Jiro Nakamura

Division of Diabetes, Department of Internal Medicine, Aichi Medical University School of Medicine, Japan

## AASD‐EASD Joint Symposium

### Saturday, May 20 9:20˜11:20 Reception Hall (West), Nagoya Congress Center Bldg. 1, 4F

#### What's new about β‐cell failure?

ChairsSusumu Seino Guy A. Rutter Kobe University Graduate School of Medicine, Japan

Section of Cell Biology and Functional Genomics, Department of Medicine, Imperial College London, UK

A‐EA1

**Selective beta‐cell dysfunction as a potential therapeutic strategy**

Pierre Maechler

Department of Cell Physiology and Metabolism, Faculty of Medicine, University of Geneva Medical Center, Switzerland

A‐EA2

**Control of pancreatic beta cell identity : roles of fuel‐sensitive kinases and transcription factors**

Guy A. Rutter

Section of Cell Biology and Functional Genomics, Department of Medicine, Imperial College London, UK

A‐EA3

**Beta cell autophagy as a therapeutic target of diabetes**

Myung Shik Lee

Department of Medicine, Division of Endocrinology and Metabolism, Sungkyunkwan University School of Medicine, Korea

A‐EA4

**Regulation of pancreatic beta cell mass from the interaction of gene‐environmental factors**

Yoshiaki Kido

Analytical Biomedical Sciences, Department of Biophysics, Kobe University Graduate School of Health Sciences, Japan

## AASD‐MDIA Joint Symposium

### Saturday, May 20 13:45˜15:45 Reception Hall (West), Nagoya Congress Center Bldg. 1, 4F

#### Genetics of diabetes in Asian

ChairsLee‐Ming ChuangDepartment of Internal Medicine, National Taiwan University Medical College, Taiwan

Hiroto FurutaThe First Department of Medicine, Wakayama Medical University, Japan

A‐MD1

**Opening remarks**

Kishio Nanjo

Wakayama Rosai Hospital, Japan

A‐MD2

**The association of ALDH2 mutation with glucose hemoestasis and renal function in human and mice**

Yi‐Cheng Chang

National Taiwan University Hospital, Taiwan

A‐MD3

**Genetics of diabetic complications**

Ronald C.W. Ma

The Chinese University of Hong Kong, Hong Kong

A‐MD4

**A population based study of monogenetic diabetes in China**

Qian Ren

Department of Endocrinology and Metabolism, Peking University People's Hospital, China

A‐MD5

**Clinical whole exome sequencing in early onset diabetes patients**

Soo‐Heon Kwak

Department of Internal Medicine, Seoul National University Hospital, Korea

A‐MD6

**Molecular analysis of suspected monogenic diabetes in children: experience of 476 cases**

Toru Yorifuji

Division of Pediatric Endocrinology and Metabolism, Children's Medical Center, Osaka City General Hospital, Japan

A‐MD7

**Genetic dissection of Japanese patients with early‐onset non type 1 diabetes and the clinical features**

Yukio Horikawa

Department of Diabetes and Endocrinology, Gifu University Graduate School of Medicine, Japan

## Symposium 1

### Friday, May 19 9:00˜11:00 Reception Hall (West), Nagoya Congress Center Bldg. 1, 4F

#### Diabetes Foot in Asia

ChairsShigeo KonoWHO‐collaborating Centre for Diabetes, Kyoto Medical Center, Japan

Zhangrong XuDiabetes Center of the 306th Hospital, Beijing, China

A‐DS1‐1

**Diabetic foot in China**

Zhangrong Xu

Diabetes Center of the 306th Hospital, Beijing, China

A‐DS1‐2

**Diabetic foot in the Philippines**

Enrico A. De Jesus

Jose R. Ryeyes Memorial Medical Center, The Philippines

A‐DS1‐3

**Diabetic foot in Cambodia**

Touch Khun

Preah Kossamak Hospital, Cambodia

A‐DS1‐4

**Diabetic Foot Care Project in AASD**

Shigeo Kono

WHO‐collaborating Centre for Diabetes, Kyoto Medical Center, Japan

A‐DS1‐5

**Diabetic foot in Vietnam**

Ta Van Binh

Hanoi Medical College, Vietnam

A‐DS1‐6

**Debridement in multidisciplinary diabetes foot ulcer care: what is the optimal frequency?**

Stephen Twigg

Department of Endocrinology, Royal Prince Alfred Hospital, University of Sydney, Australia

## Symposium 2

### Friday, May 19 13:30˜15:30 Reception Hall (West), Nagoya Congress Center Bldg. 1, 4F

#### Diabetes treatment guidelines in Asia

ChairsMoon Kyu LeeDivision of Endocrinology and Metabolism, Department of Medicine, Samsung Medical Center, Sungkyunkwan University School of Medicine, Korea

Linong Ji Peking University Diabetes Center / Department of Endocrinology and Metabolism, Peking University People's Hospital, China

A‐DS2‐1

**Glycemic targets and prevention of T2DM**

Yingying Luo

Peking University People's Hospital, China

A‐DS2‐2

**Microvascular complications in diabetes management**

Ronald C.W. Ma

The Chinese University of Hong Kong, Hong Kong

A‐DS2‐3

**Development process of clinical practice guidelines & lipid control in diabetes**

Kyung‐Mook Choi

Korea University, Korea

A‐DS2‐4

**Blood pressure management guideline in diabetes**

Daisuke Yabe

Kansai Electric Power Medical Research Institute and Kyoto University Graduate School of Medicine, Japan

A‐DS2‐5

**Medical nutrition therapy & physical activity in T2DM**

Hung‐Yuan Li

National Taiwan University Hospital, Taiwan

Co‐Sponsored by: Takeda Pharmaceutical Company Limited.

## AASD Evening Seminar

### Friday, May 19 17:20˜18:50 Reception Hall (West), Nagoya Congress Center Bldg. 1, 4F

#### Diabetes Education and Care in Asia

ChairsYutaka Seino Kansai Electric Power Hospital / Kansai Electric Power Medical Research Institute, Japan

Weiping JiaDepartment of Endocrinology and Metabolism, Shanghai Jiao Tong University Affiliated Sixth People's Hospital, Shanghai, China

AL‐ES1

**Diabetes Education and Care in China**

Linong Ji

Department of Endocrine & Metabolism, Peking University People's Hospital, Beijing, China

AL‐ES2

**Diabetes Education and Care in Japan**

Daisuke Yabe

Kansai Electric Power Medical Research Institute and Kyoto University Graduate School of Medicine, Japan

AL‐ES3

**Diabetes Education and Care in Hong Kong**

Ronald Ma

Department of Medicine and Therapeutics, The Chinese University of Hong Kong, Prince of Wales Hospital, Hong Kong

AL‐ES4

**Diabetes Education and Care in Korea**

Moon‐Kyu Lee

Sungkyunkwan University School of Medicine, Korea

AL‐ES5

**Diabetes Education and Care in Taiwan**

Wayne H.H. Sheu

Division of Endocrinology & Metabolism, Taichung Veterans General Hospital, Taiwan

AL‐ES6

**Diabetes Education and Care in Kazakhstan**

Zhanay A. Akanov

Asfendiyarov Kazakh National Medical University, Kazakhstan

Co‐Sponsored by: Nippon Boehringer Ingelheim Co., Ltd. and Eli Lilly Japan K.K.

## Educational Seminar

### Saturday, May 20 7:20–8:10 Reception Hall (West), Nagoya Congress Center Bldg. 1 4F

AASD and JDI

ChairNigishi HottaProfessor Emeritus Nagoya University / Director General Emeritus Japan Organization of Occupational Health and safety CHUBU ROSAI HOSPITAL, Japan

Yutaka Seino

Electric Power Hospital / Kansai Electric Power Medical Research Institute, Japan

Daisuke Yabe

Kansai Electric Power Medical Research Institute and Kyoto University Graduate School of Medicine, Japan

## Closing Ceremony

### Saturday, May 20 17:20˜17:35 Reception Hall (West), Nagoya Congress Center Bldg. 1, 4F

Jiro Nakamura

60th JDS President / 9th AASD President, Japan

Yutaka Seino

AASD Chair, Japan

Dato’ Ikram Shag‐bin Ismail

10th AASD President‐elect, Malaysia

